# Effects of Virtual Reality on Cardiac Rehabilitation Programs for Ischemic Heart Disease: A Randomized Pilot Clinical Trial

**DOI:** 10.3390/ijerph17228472

**Published:** 2020-11-16

**Authors:** Sara García-Bravo, Roberto Cano-de-la-Cuerda, Joaquín Domínguez-Paniagua, Raquel Campuzano-Ruiz, Estrella Barreñada-Copete, María Jesús López-Navas, Aurora Araujo-Narváez, Cristina García-Bravo, Mariano Florez-Garcia, Javier Botas-Rodríguez, Alicia Cuesta-Gómez

**Affiliations:** 1 International Doctorate School, Rey Juan Carlos University, 28922 Madrid, Spain; saragbravo@gmail.com (S.G.-B.); cristina.bravo@urjc.es (C.G.-B.); 2 Clínica de Fisioterapia Physiocare, 28026 Madrid, Spain; 3 Department of Physical Therapy, Occupational Therapy, Rehabilitation and Physical Medicine, Faculty of Health Sciences, Rey Juan Carlos University, 28922 Madrid, Spain; alicia.cuesta@urjc.es; 4 Cardiac Rehabilitation Unit, Hospital Universitario Fundación Alcorcón, Alcorcón, 28922 Madrid, Spain; jdominguez@fhalcorcon.es (J.D.-P.); rcampuzano@fhalcorcon.es (R.C.-R.); estrellabarre@gmail.com (E.B.-C.); mjlopezn@fhalcorcon.es (M.J.L.-N.); aaraujo@fhalcorcon.es (A.A.-N.); 5 Rehabilitation Service, Hospital Universitario Fundación Alcorcón, 28922 Madrid, Spain; marianotomasflorez@gmail.com; 6 Cardiology Service, Hospital Universitario Fundación Alcorcón, 28922 Madrid, Spain; jbotas@fhalcorcon.es

**Keywords:** ischemic heart disease, physical exercise, virtual reality, cardiac rehabilitation, video consoles, video games

## Abstract

(1) Background: The aim of the present study was to determine the effects of a virtual reality (VR) program, as a complementary tool to a conventional cardiac rehabilitation (CR) program in phase II of patients with ischemic heart disease compared to a conventional treatment group. (2) Methods: A single blinded randomized clinical trial was conducted. The patients were randomized to a control group (CG) or an experimental group (EG). The EG carried out a training based on VR of aerobic exercise using the XBOX ONE console and Kinect sensor. Ergometry, metabolic equivalents (METS), Functional Independence Measure, 6-min walk test (6MWT), the Short Form Health Survey-36 Questionnaire (SF-36), the Beck Depression Inventory-II, and the degree of satisfaction and adherence to treatment were used as outcome measures. (3) Results: Our results showed no statistically significant differences between the two groups. Statistical analysis within group for the EG showed statistically significant changes in the variables HR final ergometry, ergometry minutes, % ergometry, METS, final HR 6MWT, 6MWT distance, 6MWT number of laps, and for the SF-36 and Beck Depression Inventory-II. (4) Conclusion: A VR-based video game program, as an adjunct tool to a CR program, showed improvements in ergometry, METS, resistance to fatigue and health-related quality of life with excellent adherence and satisfaction perceived by patients with ischemic heart disease in phase II.

## 1. Introduction

Cardiovascular diseases (CVDs) are the leading cause of morbidity and mortality in developed countries. In Europe, CVDs are estimated to cause close to 4 million deaths annually, generating a cost of 196 billion Euros/year [1,2,3,4]. In this context, ischemic heart disease occurs due to pathophysiological changes due to an imbalance related to the demand for and the supply of oxygen in heart muscle [5,6].

Cardiac rehabilitation (CR) is defined as a set of activities necessary to ensure optimal physical, mental and social conditions in cardiac patients, which allow them to occupy as normal a place as possible in society [7]. There is a strong indication for secondary prevention including physical training; the optimization of pharmacological treatment and the control of risk factors, psychosocial support and education [8,9]. The low participation rate in CR programs is an essential factor for improvement, as well as adherence to the exercise after these programs. In this context, the European Society of Cardiology indicates the need for the creation of models that optimize outcomes in cardiac patients in CR programs [10].

To try to solve this problem, new information and communication technologies are positioning themselves as promising instruments and may be considered complementary tools to improve clinical outcomes, as well to increase motivation and adherence in CR programs [11]. Within them, virtual reality (VR) is a technology that simulates a real environment through a virtual environment in three dimensions to interact with the elements of said environment and to carry out exercise with variability, where intensity, repetition and feedback are key elements. VR is described as an additional tool to rehabilitation programs, adding a motivational factor, but its main drawback remains the high cost of these systems [12,13,14,15,16]. To solve this aspect, low-cost video game consoles, such as the XBOX^®^ One (Microsoft) console, with its Kinect^®^ accessory, or the Nintendo Wii^®^ console, with its Wii Balance Board^®^ peripheral, are being proposed as low-cost complementary tools in the CR of CVD, under training programs and physical exercise through VR (exergames) [11].

However, although VR and video game systems have captured the attention of professionals working in the field of CR, studies in this field are still limited and published protocols are very heterogeneous [17,18,19,20,21,22,23,24,25,26]. Cacau et al. [19] in a post-cardiac surgery program scheduled two sessions a day until hospital discharge. Ruivo et al. [20,25] carried out two sessions per week of 1 h duration during the 6 weeks in their phase II program. Vieira et al. [17,18,23] carried out their protocol for 6 months with three sessions a week of VR, while recommending that they carry out a 30-min walking program each day for the remaining days. The duration of each session could be increased according to the individual’s needs (10 min of warm-up followed by 20 to 25 min of strength work and 35 to 45 min of resistance, ending with 6 min of stretching). Klompstra et al. [21] applied 20-min sessions daily that could be increased if the patient was not fatigued. Jaarsma et al. [24] applied 30 min of exercise a day plus physical activity counseling for 12 months. Finally, Serber et al. [26] proposed a 60-min program per session with three sessions per week for 12 weeks.

Among the few publications in the scientific literature about ischemic heart disease with VR and video game systems, research seems to show that such programs could increase the functional capacity, motivation and adherence of patients to CR programs [11]. However, there is no consensus regarding the outcome measures to be studied, the number of sessions, the protocol to be followed or the technology to be used, since there is great heterogeneity in the scientific literature [11]. Therefore, despite the potential usefulness of the use of VR and video games, there is a need to determine the ideal intervention protocol, as well as its effect on the main short- and medium-term outcome measures in CR programs.

The aim of this pilot research was to determine the effects of a VR program and video games, as complementary tools to a conventional rehabilitation program, in the CR of patients with ischemic heart disease in phase II, compared to a conventional treatment group in terms of ergometry, metabolic equivalents, functionality, aerobic capacity and endurance, quality of life, depression, satisfaction and adherence. Our initial hypothesis is that our structured protocol, using VR and video game systems, could be a complement to conventional therapy and produce improvements in ergometric variables, METS, fatigue resistance, as well as in quality of life and perceived depression, with excellent adherence and perceived satisfaction.

## 2. Materials and Methods

### 2.1. Design

A single blind randomized clinical trial (observer-blinded) was performed. All participants with ischemic heart disease came from the CR Unit of Hospital Universitario Fundación Alcorcón (Madrid, Spain). The CONsolidated Standards of Reporting Trials, CONSORT checklist [27] was followed to guarantee the methodological quality of this work.

This project was approved by the Research Ethics Committee of the Hospital Universitario Fundación Alcorcón (11032019CEIm), prior to the start of the project. The ethical principles for medical research in humans of the Declaration of Helsinki were followed. All participants were informed of the objectives of the present study and provided signed informed consent. Finally, the present work was registered with Clinical Trials (NCT04166422).

### 2.2. Participants

All subjects were recruited from the Cardiology Service of Hospital Universitario Fundación Alcorcón (Madrid, Spain). Patients had to meet the following inclusion criteria: subjects of both sexes, >18 years of age, diagnosis of ischemic heart disease by the Cardiologist, low risk stratification, level I on the Canadian Cardiovascular Society scale and in the Goldman Physical Activity Scale, ≥7 METS in conclusive ergometry, ejection fraction >50% and with the indication of cardiac rehabilitation [8]. All patients had to be hemodynamically stable and without recent instability from other non-cardiologic comorbidities.

The exclusion criteria of the present study were absence of maximum and stopped ergometry due to symptoms, pregnancy, the presence of a pacemaker, conditions that hindered the use of VR such as the presence of visual deficiencies or hearing, learning disabilities, cognitive impairment, psychiatric pathology or the use of support products for ambulation or standing, presence of other serious neurological, musculoskeletal or lung diseases, uncompensated metabolic disorders, previous cardio-respiratory arrest or history of photosensitive epilepsy for video game use.

The patients were randomly assigned by means of the QuickCalcs application of GraphPad Software^®^ (GraphPad Inc, San Diego, CA, USA) to two study groups: a control group (CG), which received a conventional CR program, and an experimental group (EG), which received a combined program of conventional CR together with physical exercise through VR and video games.

A total of 26 participants were initially recruited (CG = 18 and EG = 18) at the study onset. Twenty participants completed all sessions and assessments and were included for the statistical analysis. Six subjects did not fulfill the rehabilitation program (CG = 3 and EG = 3) due to COVID-19 pandemic, so CR Unit must close all clinical services.

### 2.3. Procedure

Once in the CR Unit, all participants in the CG followed the conventional CR protocol of the Fundación Alcorcón University Hospital. The EG participants followed a CR protocol in combination with the use of VR and video games using the Microsoft XBOX^®^ One console (Microsoft company, Albuquerque, NM, USA) and its Kinect 2^®^ peripheral (Microsoft Company, Albuquerque, NM, USA). Both groups received the same number of sessions and treatment times in the same CR Unit between September 2019 and March 2020.

The XBOX game console features an ATI Xenos graphics processing unit, 10 MB, 512 MG RAM, three USB 2.0 ports, two memory card slots and four wireless controllers. The Kinect^®^ motion sensor offers (1) infrared sensors that collect depth data in relation to the room and establish the distance at which the subject is and its position and allow calculating its relative size, (2) an RGB camera that complements facial and body recognition and (3) multi-array microphones capable of detecting different voices and extracting ambient sound. Through software, Kinect generates a 3D map of the image that it receives in its visual field, creating a digital skeleton or avatar, so that the subject can interact with the device, allowing it to capture 3D movements of the entire body. It allows you to use your own body as a remote control for games.

**Figure 1:**
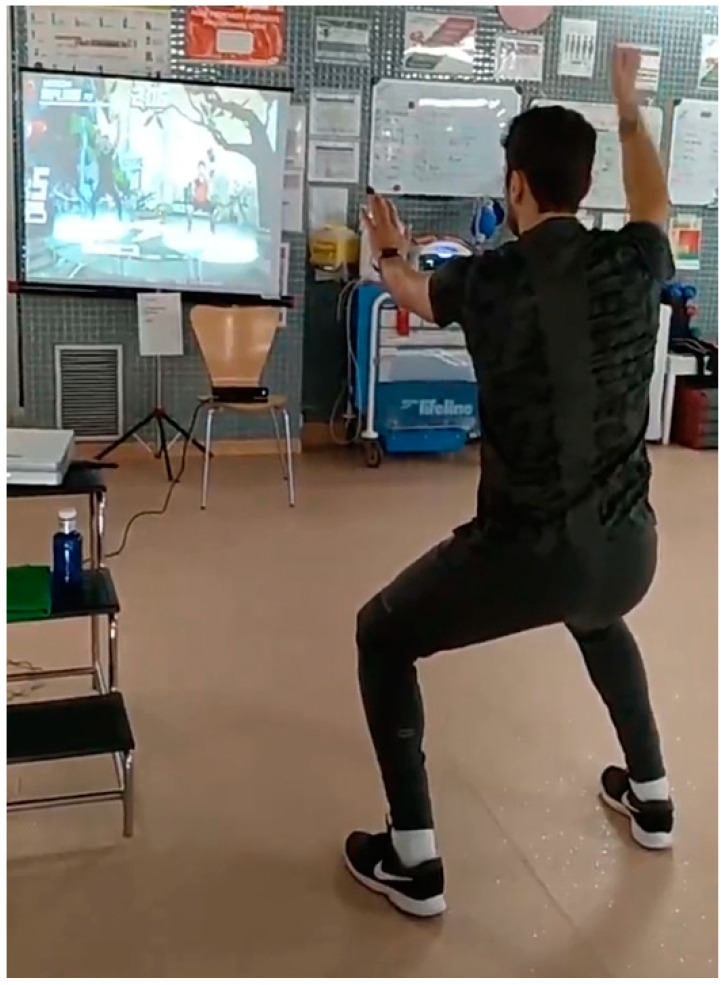
Patient performing the experimental protocol.

**Figure 2:**
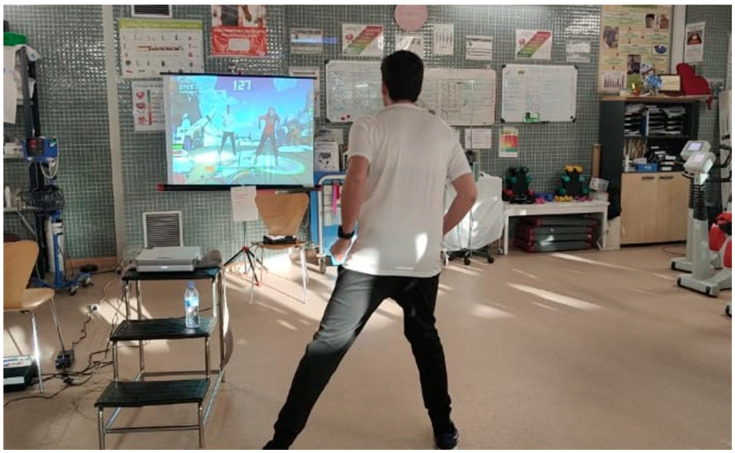
Patient performing the experimental protocol.

Currently, there is no application or software on the market specifically designed for ischemic heart disease patient rehabilitation use for the XBOX ONE console. For this reason, the VR treatment program was developed by the research team with clinical and research experience in the field of rehabilitation technologies, based on their commercial Kinect Shape Up^®^ software (Microsoft Company, Albuquerque, NM, USA) (Table 1). The VR gaming protocol proposed aerobic activities, such as dodging objects, avoiding obstacles, imitating postures (Figure 1), squats, steps (Figure 2), among others, based on a previous systematic review [11]. The selected games were repeated throughout the program, with increasing difficulty, as well as the gradual incorporation of other games. Their intensity and duration were adapted according to the limits of heart rate and the sensation of effort for each patient.

All participants received two sessions a week for 8 weeks (total 16 sessions). The time of each session was 60 min, divided into 10 min of warm-up, 40 min of training and 10 min of cool-down or return to calm. Both groups followed the same protocol for warm-up and cool-down, while aerobic training varied by group. In the CG, the training consisted of resistance exercises (treadmill for 30 min) and limb strength with weights of 0.5–3 kg (10 min) [8], while the EG carried out VR-based training (during 20 min), resistance exercises (endless belt) for 10 min and limb strength exercises with weights of 0.5–3 kg (10 min).

The same physical therapist supervised all interventions through the use of VR and video games, with previous experience in managing these systems. At the same time, another physiotherapist performed all of the conventional CR interventions in the CR unit. The prescribed heart rate for training was 75 and 85% of the maximum heart rate (HR) reached in the baseline ergometry, applying to the first and second month of treatment [8], respectively, for both groups. HR during rehabilitation session was monitored by telemetry (Sana-Sprint PLUS Ergosana Rehab System) to check the correct achievement the heart rate for training. All treatments were also supervised by a cardiologist expert in CR and nursing staff, taking into account the degree of subjective perception of effort of each patient.

### 2.4. Outcome Measures

Before the start of the CR program and after finishing the intervention program for both groups, the following outcome measures were evaluated:

- Ergometry. In the present study, a treadmill (Bruce protocol) was used as an ergometric test, in three-minute stages. At each stage, both the speed and slope of the treadmill increased, causing an increase in workload. Exceeding 85% maximum HR (220-Patient age) as submaximal HR (>85%) was used as a theoretical guide. The Bruce protocol has a maximum duration of 21 min, with a recovery phase of 8–10 min, until the parameters evaluated are normalized, with the first two minutes being especially important. The ergometry ended with the following assumptions: reaching 85% of the maximum HR in the presence of physical exhaustion, appearance of symptoms, perception of a certain effort on the Börg Scale or altered electrical activity in the electro-cardiogram [28]. The final HR of the ergometry, the time in minutes of the ergometry and the % complete of the ergometry (considering as conclusive >85% estimated HR) were recorded as variables.
- Metabolic Equivalents (METS). The equivalence table was used for the transformation formula from Watts to METS, in order to estimate the VO_2_max (1 MET equals 3.5 mL/kg/min), proposed by the Spanish Society of Cardiology [29].
- Functional Independence Measure. This is the most accepted functional evaluation measure in the field of rehabilitation. It values six functional areas: self-care, toileting, transfers, locomotion, communication and awareness of the outside world. The first four include 13 items and comprise motor function. The areas related to communication and awareness with the outside world comprise five items and refer to the cognitive sphere. The total score can vary between 7 (total dependence) and 126 (total independence) [30,31].
- 6-min walk test (6MWT). To carry out this test, a closed and marked corridor with a length equal to 30 m was available. It was carried out by going around this section, delimited by indicators on the ground. These signs were placed at a distance of 29 m from each other, leaving 0.5 m at each end for the patient to rotate. Following the international recommendations, the patient walked in the company of the examiner, carrying a portable pulse oximeter that recorded both HR and O_2_ saturation (% O_2_) every minute. Before starting this, the patient was reminded of the idea of traveling as far as possible in 6 min, being able to change the rhythm or stop if needed. The distance traveled in meters was recorded [32]. The final HR at 6MWT, the recovery HR at two minutes, the average HR, the maximum distance traveled in meters, the total number of laps, as well as the maximum HR were recorded as outcome measures.
- Short Form Health Survey-36 Questionnaire (SF-36). The SF-36 is an assessment instrument used by a larger number of authors in the assessment of health-related quality of life in patients with heart disease. It is made up of 35 scoring items, divided into 8 dimensions: Physical Function, Physical Role, Emotional Role, Social Function, Mental Health, General Health, Body Pain and Vitality. It also contains an additional item that is not part of any dimension and which measures the declared evolution of health [33,34].
- Beck-II Depression Inventory. This questionnaire was used to detect and assess the severity of depression in the sample. It is based on a self-administered questionnaire with 21 items that are answered on a four-point Likert-type scale (from 0 to 3), except for items 16 (changes in sleep pattern) and 18 (changes in appetite) which contain seven categories. The minimum and maximum scores on the test are 0 (no depression) and 63 (severe depression) [35].
- Degree of satisfaction with the treatment. The Client Satisfaction Questionnaire was used to assess satisfaction with the health service provided to both groups. This is a self-administered questionnaire, consisting of eight questions that assess the level of satisfaction in relation to the care and quality of the service received, as well as the degree of compliance with the patient’s expectations prior to the intervention. The responses are coded from 1–4, with the total score of the questionnaire being 32 points, where higher values indicate greater satisfaction with the treatment received [36].
- Additionally, the EG completed a specific questionnaire regarding treatment with VR technology and video games, designed and used by the research group based on previous research in the field of rehabilitation using technologies. The questionnaire is made up of 18 items that assess the degree of satisfaction in the following dimensions: technical quality of the equipment (four items), ease of use of video games (five items), compliance with the program and applicability (seven items) and degree of satisfaction or complacency (two items). The responses are established based on a Likert-type scale of 1–5 points, with alternative directionality of the responses to avoid stereotyped responses. Regarding the interpretation of the results, the maximum possible score is 90 points [14,15,16].
- Adherence. The percentage of attendance to both treatment modalities was registered, as well as the presence of adverse effects (such as nausea, vomiting, headache, dizziness, muscle aches or general pain) in both groups.

All participants were evaluated by the same rehabilitative doctor and physical therapist, experts in CVD care in CR units, who were not involved in the intervention, at the beginning and after the completion of phase II of CR in the hospital.

### 2.5. Statistical Analysis

Statistical analysis was performed using the SPSS statistical software system (SPSS Inc., Chicago, IL, USA; v24). The Kolmogorov–Smirnov test and the Shapiro–Wilk test were used to analyze all of the data for normality of distribution. Since the sample did not follow a normal distribution, the Mann–Whitney test for unrelated samples and the Wilcoxon test for related samples were used to compare the variables. Statistical analysis was performed with a confidence level of 95% and significant values were considered as *p* < 0.05. The mean and standard deviation of the parameters were used to calculate the effect size for the comparisons using Cohen’s d statistic. Mean differences of 0.2, 0.5 and 0.8 standard deviations are considered small, moderate and large effect sizes, respectively. No sample size calculation was performed as this randomized clinical trial was considered a pilot study.

## 3. Results

### 3.1. Sociodemographic Data

**Figure 3:**
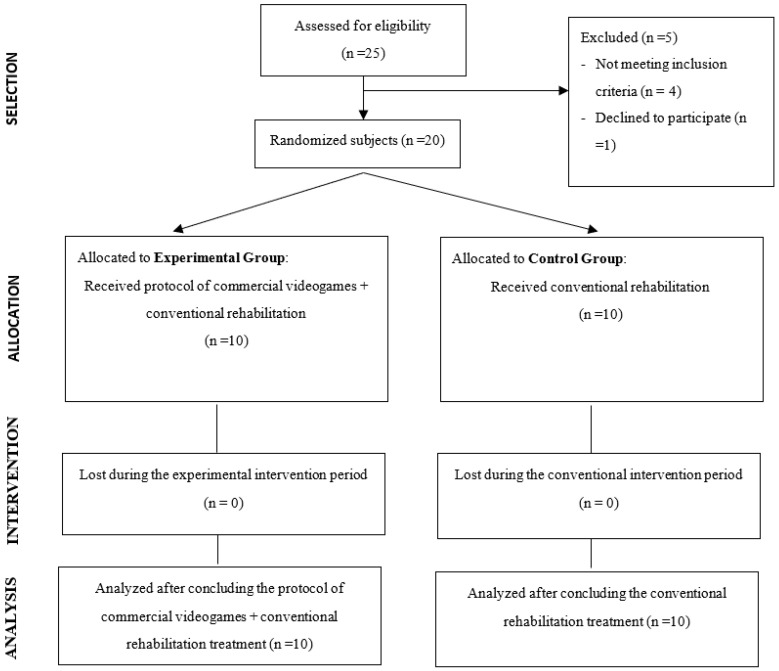
Flow chart.

Twenty participants completed the study, with a mean age of 51.20 (±8.82) years, mean weight of 84.89 (± 16.02) kg, mean height of 171.85 (±7.69) cm and average body mass index of 28.64 (±4.22) kg/cm. These patients were randomized into two groups of 10 participants, respectively (Figure 3). The EG (*n* = 10) presented a mean age of 48.70 (±6.66) years, mean weight of 75.23 (±6.91) kg, mean height of 161.00 (±7.95) cm and an average mass index body of 26.55 (±3.19). The CG presented a mean age of 53.7 (±10.30) years, mean weight of 94.56 (±16.92) kg, mean height of 175.00 (±6.28) cm and a mean body mass index of 30.73 (±4.21). No statistically differences regarding age (*p* = 0.289), weight (*p* = 0.065), height (*p* = 0.051) and the average mass index body (*p* = 0.059) were observed between the control and experimental groups at the beginning of the study.

### 3.2. Intra-Group and Inter-Group Statistical Analysis

The intra-group statistical analysis (Table 2) showed statistically significant changes in the EG after treatment for the following variables: For final HR ergometry (*p* = 0.028) and ergometry minutes (*p* = 0.049), the effect size was moderate (>0.50); for % ergometry (*p* = 0.021), the effect size was moderate (>0.50); for METS (*p* = 0.050), the effect size was moderate (>0.50); for final 6MWT FC (*p* = 0.028), the effect size was moderate (>0.50); for 6MWT distance (*p* = 0.005), the effect size was large (>0.80); and for 6MWT number of turns (*p* = 0.005), the effect size was large (>0.80). Of the variables analyzed in the SF-36 Health questionnaire, statistically significant changes were observed in the evaluations after treatment for General Health (*p* = 0.049), where the effect size was moderate (>0.50); for vitality (*p* = 0.011), the effect size was moderate (>0.50); for social function (*p* = 0.010), the effect size was moderate (>0.50); the effect size was large for social function (>0.80), and for the declared evolution of health (*p* = 0.024), the effect size was moderate (>0.50). In addition, statistically significant changes in the level of depression were observed (*p* = 0.012), with a moderate effect size (>0.50).

Statistically significant changes were observed in the CG (Table 3) after treatment in the following variables: Ergometry minutes (*p* = 0.017), where the effect size was moderate (>0.50), for METS (*p* = 0.008), the effect size was moderate (>0.50); for 6MWT distance (*p* = 0.005), the effect size was moderate (>0.50), and for 6MWT number of turns (*p* = 0.005), the effect size was moderate (>0.50). The variables of the SF-36 that showed statistically significant changes after treatment were physical function (*p* = 0.026) and body pain (*p* = 0.036)), where the effect size was moderate (>0.50).

Regarding the inter-group analysis (Table 4), no statistically significant differences were observed between the two groups.

In the Client Satisfaction Questionnaire, the EG obtained an average score of 31.60 (±0.96) and the CG 30.70 (±2.86). Of the eight items on the questionnaire, the EG obtained the maximum score for items 3, 4, 5, 7 and 8. In the CG, the maximum score was not observed for any of the items on the questionnaire. However, it should be noted that none of the subjects showed disagreement or dissatisfaction regarding the remaining questions (Table 5).

Related to the satisfaction questionnaire with the VR and video games program, the EG presented a high degree of satisfaction, obtaining an average score of 82.50 (±8.33) out of 90 (Table 6). Finally, 100% adherence to treatment was observed in the two intervention groups. No adverse effect was recorded with the control intervention or with the intervention combined with VR technology.

## 4. Discussion

To the best of our knowledge, this is the first randomized clinical trial conducted on the use of VR and video games in ischemic heart disease, phase II, in a hospital setting. Our results point out that conventional CR in combination with physical exercise through a protocol using the Microsoft XBOX^®^ One console and its Kinect 2^®^ peripheral produced improvements in ergometric variables, METS, fatigue resistance, as well as in quality of life and perceived depression in the EG. In addition, adherence to treatment was excellent, as was perceived satisfaction with the applied protocol.

Currently, CR programs are considered safe and effective (level of evidence 1a: systematic reviews with homogeneity of randomized controlled trials) by the American College of Cardiology and the American Heart Association, with a higher level recommendation (A) than physical exercise as the basis of CR, as it reduces the morbidity and mortality associated with coronary heart disease, with a decrease in complications and mortality close to 40% in low-risk patients after myocardial infarction [37]. Likewise, with this type of intervention, the quality of life related to health improves significantly (level 1A). Furthermore, it is known that the risk of exercise is perfectly acceptable, given the benefits it provides. Other authors conclude that the cost-effectiveness and cost-benefit ratios of CR are, today, the most favorable of all of the treatments and interventions practiced in heart disease management. It is also suggested that the benefits of these programs diminish over time, so it is important to expand the range of proposals that ensure adherence and satisfaction with these programs, as they will be useful for monitoring compliance and/or the prevention of modifiable risk factors [38]. Therefore, it should be noted that, despite the fact that no statistically significant differences were reached between the intervention groups, a greater number of intra-group variables were presented with statistically significant changes in favor of EG in comparison with CG, understanding the latter as the conventional treatment of reference and the highest level of scientific evidence and degree of recommendation in the scientific literature at this moment.

Phase II of CR should start as soon as possible and last for approximately two to three months. It can be done in the hospital or in specialized units under medical supervision. In this phase, the patient must actively work with controlled aerobic physical exercises, including stretching and strength training, then move to cycle ergometer or treadmill training and subsequently to the cooling phase [8]. However, this conventional phase II approach is not without limitations, such as a lack of adherence and motivation on the part of patients [10,11]. For this reason, the use of new therapeutic strategies, such as VR and video games in the CR of ischemic heart disease, could be a contributing tool of interest.

Two recent systematic reviews [11,39] demonstrate the main methodological limitations of previous publications on the use of VR and video games as a therapeutic tool in the approach to CVD in CR units. The clinical heterogeneity of the included patients, with different age ranges, has been highlighted, and VR has been applied in all phases of CR. It should be noted that all of the ischemic heart disease patients included in this study were in phase II of CR, having completed phase I in the same hospital.

According to the types and tools of VR, the most commonly used systems in previous studies have been low-cost consoles, such as Microsoft XBOX^®^ with its Kinect^®^ peripheral and the Nintendo Wii^®^, both reported as the most accessible systems in the acquisition and management for patients with CVD [11,39]. In most studies, VR is used as a coadjuvant to conventional cardiac rehabilitation [11]. In the present study, the VR intervention was combined with a conventional CR protocol. The commercial software and protocol specifically designed by the research team provoked, in a constant way and in real-time, adapted neuromuscular responses and at the cardio-respiratory level until reaching the established training heart rate and was always monitored and controlled by the same physical therapist in the context of a CR unit.

There is considerable heterogeneity in the scientific literature regarding VR intervention protocols [11]. Our protocol was based on previous systematic reviews [11,39], as well as the organizational and material requirements available in the hospital in which the treatments were carried out, based on two sessions a week for 8 weeks, with a total of 16 established sessions. The time per session was 60 min, divided into 10 min of warm-up, 40 min of training, split into physical exercise using VR (20 min), resistance exercise with an endless treadmill (10 min) and limb strength with 0.5–3 kg weights (10 min), followed by 10 min of cooling down.

According to previous studies [39,40,41], the hemodynamic and physiological effects of VR are comparable to CR, but exercise intensity should be mild to moderate to achieve results, with the risks described in adults and older adults with CVD being minimal [11]. Some authors [42] indicate that the metabolic rates that can be consumed by performing VR in healthy population through the Wii^®^ and the Wii Sports software is variable (between 1.3 and 5.6 METS). These values correspond to the energy expenditure of a slow gait or very fast gait (for 1.3 METS and 5.6 METS, respectively). Bosch et al. [43] assessed whether 30 min of boxing with the Wii^®^ produced cardiorespiratory benefits in healthy young people from 23–27 years old compared to a treadmill, concluding that VR promoted adequate aerobic activity and could be used as a viable alternative in RC. In our work, the EG showed improvements in various variables of the ergometry (final HR of the ergometry, minutes of the test, % ergometry), as well as in the METS and the 6MWT (final HR, total distance traveled and number of laps). In the CG, significant values were reached for the time variables in minutes of the ergometry, METS, 6MWT distance and 6MWT number of turns, reinforcing the hypothesis that the combined treatment with VR offers equal or superior improvements, without any significant differences between the two treatment modalities.

Previous publications [11,39] point to the improvement of motivational factors as an advantage of VR and video games in the treatment of CVD. In the present study, the EG subjects reported that it was a more interactive and motivating form of treatment, which could be related to the excellent degree of adherence achieved (100%). The scores on the Client Satisfaction Questionnaire were slightly higher in favor of the EG, reaching the highest score on the items of recommendation, satisfaction, confidence and problem solving. In relation to the specific satisfaction with the VR program, the patients selected the following as the best valued aspects (≥4.70 points): accessibility, the total number of sessions, the care received, personalized attention and general satisfaction with the VR. These data seem to be in line with the opinion of health professionals who use VR in physical training programs [44] and indicate that these systems are easy to use, safe and can improve treatment compliance. However, it is indicated that this form of treatment is not applicable for all CVDs, so inclusion criteria, monitoring, supervision and a proper design of protocols are key in CR with VR.

Despite not finding significant differences between the groups, the subjects of the EG experienced improvements in items of the SF-36 such as general health, vitality, social function and in the declared evolution of health, as well as in the level of depression assessed. It should be noted that the EG had slightly higher scores than the pre-treatment CG. No significant intra- or inter-group differences were found for functionality, since both groups started from a maximum score, as well as in the psychosocial variables. Future work should take these findings into account by using functionality questionnaires based on the observation of tasks, as well as by implementing follow-up evaluations and adherence to physical exercise through VR in phase III, in order to objectify their potential effects on mood, depression and anxiety.

The objective of the present study was to explore the effects of VR and video games plus conventional treatment in ischemic heart disease, phase II, in a hospital setting. Our results demonstrate that VR could be incorporated to CR programs. Future studies taking into account our results should explore a comparison between VR isolated and conventional treatment in patients with ischemic heart disease in phase II.

The present study presents limitations. Firstly, the small sample size makes future work necessary to corroborate our findings. Further, the lack of significant differences between groups in terms of sociodemographic data (age, weight, height and BMI) at the beginning of the study could be due to a small sample size and it might be insufficient to detect a difference between groups. Second, the results cannot be extrapolated to all CVDs, high-risk patients, or all phases of CR, so the results should be interpreted with caution. Moreover, an older population should be included in future studies because age could influence motivation and compliance results. Thirdly, non-specific CR software was used, despite monitoring of the training heart rate. Fourthly, a non-validated measure of motivation assessment was used in this research. Finally, long-term follow-up data are not yet available since patients have been discharged from the CR unit, so future work should implement short-, medium- and long-term follow-up evaluations, mainly regarding adherence to the practice of physical exercise in phase III.

## 5. Conclusions

A VR and video game program, as an adjunct tool to a conventional CR program, produced improvements in ergometry, METS estimation, resistance to fatigue, quality of life and depression, with excellent adherence and satisfaction perceived by patients with ischemic heart disease in phase II. There were no statistically significant differences in relation to the conventional intervention, so these could become alternatives to regular programs in patients with low-risk ischemic heart disease.

Our low-cost VR protocol could be an alternative to the fixed equipment of a CR unit, considering it is a safe intervention. Future studies should focus on adherence to physical exercise in patients with IC in phase III of CR under this type of intervention with VR by means of follow-up evaluations.

## Figures and Tables

**Table 1:** Virtual reality protocol and video games.

**1 and 2 weeks**	120″ relative rest
	Slalom of extensions (4:00′)
	180″ relative rest
	Squats to the moon (1:30′)
	180″ relative rest
	Slalom of extensions (4:00′)
	150″ relative rest
**3 and 4 weeks**	Slalom of extensions (4:00′)
	150″ relative rest
	Squats to the moon (1:30′)
	120″ relative rest
	Acrobatic Race (1:30′)
	150″ relative rest
	Slalom of extensions (4:00′)
	120″ relative rest
**5 and 6 weeks**	Slalom of extensions (4:00′)
	90″ relative rest
	Squats to the moon (1:30′)
	90″ relative rest
	Snowstorm (4:00′)
	120″ relative rest
	Slalom extensions (4:00′)
	60″ relative rest
**7 and 8 weeks**	Slalom of extensions (4:00′)
	60″ relative rest
	Fight stomp (4:00′)
	60″ relative rest
	Acrobatic Race (1:30′)
	60″ relative rest
	Squats to the moon (1:30′)
	60″ relative rest
	Slalom of extensions (4:00′)
	60″ relative rest

Kinect Shape Up^®^ Software (Microsoft Company, Albuquerque, NM, USA) Gaming Protocol.

**Table 2:** Comparison outcome measures experimental group (intra-group analysis).

Variable	Experimental Group			
	Pre	Post	Intra-Group Analysis *p* Value	Cohen’s d
	Median (Interquartile Range)			
**Final HR ergometry**	138.00 (35.00)	155.00 (42.00)	0.028 *	0.714
**Ergometry minutes**	9.00 (2.66)	11.11 (2.98)	0.049 *	0.664
**% ergometry**	82.00 (14.00)	92.00 (18.50)	0.021 *	0.746
**METS**	10.00 (3.00)	13.30 (4.00)	0.050 *	0.714
**FIM**	126.00 (0)	126.00 (0)	1.000	0
**Final 6MWT FC**	89.00 (17.50)	92.00 (29.50)	0.028 *	0.495
**6MWT FC recovery**	72.00 (17.00)	66.00 (24.50)	1.000	0.013
**6MWT FC average**	89.00 (20.50)	88.00 (27.50)	0.125	0.391
**6MWT distance**	457.80 (132.00)	513.00 (117.00)	0.005 *	0.837
**6MWT number of laps**	7.63 (2.20)	8.55 (1.95)	0.005 *	0.837
**6MWT maximum FC**	174.00 (8.00)	174.00 (8.00)	0.157	0.048
**SF-36. Physical Function**	90.00 (17.50)	100.00 (15.00)	0.104	0.596
**SF3-6. Physical role**	100.00 (62.50)	100.00 (50.00)	0.157	0.205
**SF-36. Body ache**	72.00 (17.00)	84.00 (54.00)	0.498	0.035
**SF-36. General health**	72.00 (20.00)	77.00 (21.50)	0.049 *	0.498
**SF-36. Vitality**	65.00 (32.50)	80.00 (37.50)	0.011 *	0.618
**SF-36. Social function**	63.00 (50.50)	100.00 (18.75)	0.010 *	0.841
**SF-36. Emotional role**	67.00 (83.50)	100.00 (66.70)	0.680	0.371
**SF-36. Mental health**	56.00 (44.00)	84.00 (46.00)	0.196	0.413
**SF-36. Declared evolution of health**	4.00 (2.00)	3.00 (3.00)	0.024 *	0.721
**Beck-II Depression Inventory**	12 (27.00)	11.00 (30.00)	0.012 *	2.158

HR: heart rate; METS: metabolic equivalents; FIM: Functional Independence Measure; 6MWT: 6-min walk test; SF-36: Short Form Health Survey-36 Questionnaire. The data has been expressed as median and interquartile range. * *p* value < 0.05 using the Wilcoxon test for related samples.

**Table 3:** Comparison outcome measures control group (intra-group analysis).

Variable	Control Group			
	Pre	Post	Intra-Group Analysis *p* Value	Cohen’s d
	Median (Interquartile Range)			
**Final HR ergometry**	143.00 (12.75)	144.00 (11.75)	0.813	0.163
**Ergometry minutes**	9.46 (2.89)	9.83 (3.11)	0.017 *	0.607
**% ergometry**	86.00 (7.00)	86.50 (5.00)	0.858	0.043
**METS**	11.50 (3.00)	12.65 (3.23)	0.008 *	0.574
**FIM**	126.00 (0)	126.00 (0)	1.000	0
**Final 6MWT FC**	92.00 (15.50)	96.00 (13.50)	0.059	0.772
**6MWT FC recovery**	67.00 (15.50)	71.00 (16.75)	0.241	0.508
**6MWT FC average**	89.00 (14.25)	91.50 (12.00)	0.078	0.778
**6MWT distance**	462,30 (65.40)	557,40 (70.35)	0.005 *	0.757
**6MWT number of laps**	7.70 (1.09)	9.29 (1.17)	0.005 *	0.316
**6MWT maximum FC**	167,50 (16.25)	167.00 (17.00)	0.180	0.438
**SF-36. Physical Function**	85.00 (22.50)	95.00 (5.00)	0.026 *	0.787
**SF3-6. Physical role**	100.00 (100.00)	100.00 (25.00)	0.066	0.642
**SF-36. Body ache**	80.00 (28.00)	100.00 (27.00)	0.036 *	0.640
**SF-36. General health**	62.00 (33.50)	77.00 (40.00)	0.259	0.477
**SF-36. Vitality**	70.00 (50.00)	75.00 (27.50)	0.106	0.661
**SF-36. Social function**	100.00 (43.50)	100.00 (12.25)	0.115	0.659
**SF-36. Emotional role**	100.00 (50.00)	100.00 (50.00)	1.000	0.033
**SF-36. Mental health**	72.00 (42.00)	88.00 (18.00)	0.075	0.680
**SF-36. Declared evolution of health**	3.00 (2.00)	2.00 (1.50)	0.132	0.675
**Beck-II Depression Inventory**	7.00 (8.00)	3.00 (7.00)	0.123	0.638

HR: heart rate; METS: metabolic equivalents; FIM: Functional Independence Measure; 6MWT: 6-min walk test; SF-36: Short Form Health Survey-36 Questionnaire. The data has been expressed as median and interquartile range. * *p* value < 0.05 using the Wilcoxon test for related samples.

**Table 4:** Comparison of outcome measures between the experimental group and the control group (intergroup analysis).

Variable	Experimental Group			Control Group			Experimental Group vs. Control Group	
	Median (Interquartile Range)		Mean Difference	Median (Interquartile Range)		Mean Difference		
	Pre	Post		Pre	Post		Pre *p* Value	Post *p* Value
**Final HR ergometry**	138.00 (35.00)	155.00 (42.00)	12.80	143.00 (12.75)	144.00 (11.75)	−1.30	0.307	0.225
**Ergometry minutes**	9.00 (2.66)	11.11 (2.98)	1.73	9.46 (2.89)	9.83 (3.11)	0.99	0.880	0.364
**% ergometry**	82.00 (14.00)	92.00 (18.50)	7.30	86.00 (7.00)	86.50 (5.00)	1.30	0.288	0.570
**METS**	10.00 (3.00)	13.30 (4.00)	2.06	11.50 (3.00)	12.65 (3.23)	1.11	0.692	0.401
**FIM**	126.00 (0)	126.00 (0)	0.00	126.00 (0)	126.00 (0)	0.00	0.317	0.317
**Final 6MWT FC**	89.00 (17.50)	92.00 (29.50)	5.70	92.00 (15.50)	96.00 (13.50)	6.80	0.910	0.472
**6MWT FC recovery**	72.00 (17.00)	66.00 (24.50)	0.40	67.00 (15.50)	71.00 (16.75)	3.70	0.405	0.667
**6MWT FC average**	89.00 (20.50)	88.00 (27.50)	3.90	89.00 (14.25)	91.50 (12.00)	6.30	0.520	0.725
**6MWT distance**	457.80 (132.00)	513.00 (117.00)	76.50	462.30 (65.40)	557.40 (70.35)	97.20	0.623	0.970
**6MWT number of laps**	7.63 (2.20)	8.55 (1.95)	1.27	7.70 (1.09)	9.29 (1.17)	1.62	0.623	0.970
**6MWT maximum FC**	174.00 (8.00)	174.00 (8.00)	−0.20	167.50 (16.25)	167.00 (17.00)	−0.30	0.289	0.307
**SF-36. Physical Function**	90.00 (17.50)	100.00 (15.00)	7.77	85.00 (22.50)	95.00 (5.00)	12.77	0.823	0.925
**SF3-6. Physical role**	100.00 (62.50)	100.00 (50.00)	5.55	100.00 (100.00)	100.00 (25.00)	22.22	0.844	0.576
**SF-36. Body ache**	72.00 (17.00)	84.00 (54.00)	2.44	80.00 (28.00)	100.00 (27.00)	18.33	0.964	0.227
**SF-36. General health**	72.00 (20.00)	77.00 (21.50)	8.66	62.00 (33.50)	77.00 (40.00)	7.55	0.755	0.658
**SF-36. Vitality**	65.00 (32.50)	80.00 (37.50)	11.11	70.00 (50.00)	75.00 (27.50)	12.77	0.790	0.374
**SF-36. Social function**	63.00 (50.50)	100.00 (18.75)	27.83	100.00 (43.50)	100.00 (12.25)	12.61	0.096	0.599
**SF-36. Emotional role**	67.00 (83.50)	100.00 (66.70)	11.06	100.00 (50.00)	100.00 (50.00)	0.00	0.288	0.693
**SF-36. Mental health**	56.00 (44.00)	84.00 (46.00)	6.66	72.00 (42.00)	88.00 (18.00)	11.11	0.155	0.105
**SF-36. Declared evolution of health**	4.00 (2.00)	3.00 (3.00)	−1.00	3.00 (2.00)	2.00 (1.50)	−0.66	0.135	0.455
**Beck-II Depression Inventory**	12 (27.00)	11.00 (30.00)	8.55	7.00 (8.00)	3.00 (7.00)	3.55	0.084	0.424
**CSQ-8**		32.00 (0.25)			32.00 (1.50)			0.551

HR: heart rate; METS: metabolic equivalents; FIM: Functional Independence Measure; 6MWT: 6-min walk test; SF-36: Short Form Health Survey-36 Questionnaire; CSQ-8: Client Satisfaction Questionnaire. The data has been expressed as median and interquartile range. *p* value < 0.05 using the Mann–Whitney test for non-related samples.

**Table 5:** Degree of satisfaction with the treatment client satisfaction questionnaire.

Variable	Experimental Group	Control Group
**1. Quality of service**	3.9 (0.31)	3.8 (0.42)
**2. Type of service**	3.9 (0.31)	3.70 (0.48)
**3. Needs covered**	4 (0)	3.80 (0.63)
**4. Would you recommend to a friend**	4 (0)	3.8 (0.42)
**5. Amount of aid**	4 (0)	3.90 (0.31)
**6. Solve problems**	3.8 (.42)	3.90 (0.31)
**7. General satisfaction**	4 (0)	3.90 (0.31)
**8. Would you go back again**	4 (0)	3.90 (0.31)
**Total score**	31.60 (0.96)	30.70 (2.86)

Data has been expressed as mean and standard deviation.

**Table 6:** Degree of satisfaction with the VR and videogames treatment.

Variable	Experimental Group
**1. Accessibility**	4.80 (0.42)
**2. Ease of handling**	4.60 (0.69)
**3. Fun**	4.30 (0.82)
**4. Graphics and music**	4.20 (0.78)
**5. Duration of the program**	4.50 (0.70)
**6. Duration of the program**	4.30 (0.94)
**7. Understanding**	4.60 (0.69)
**8. Objective-result**	4.60 (0.51)
**9. Progression difficulty**	4.60 (0.51)
**10. Total number of sessions**	4.70 (0.67)
**11. Availability**	4.60 (0.69)
**12. Attention received**	4.90 (0.31)
**13. Clarity in explanations**	5.00 (0)
**14. Personalized attention**	4.90 (0.31)
**15. Possibility to objectify results**	4.50 (0.70)
**16. Possibility of transferring games to activities of daily life**	4.30 (0.82)
**17. Compliance with expectations**	4.40 (0.84)
**18. General satisfaction**	4.70 (0.48)
**Total score**	82.50 (8.33)

Data has been expressed as mean and standard deviation.
